# On the advantages and disadvantages of choice: future research directions in choice overload and its moderators

**DOI:** 10.3389/fpsyg.2024.1290359

**Published:** 2024-05-09

**Authors:** Raffaella Misuraca, Ashley E. Nixon, Silvana Miceli, Giovanni Di Stefano, Costanza Scaffidi Abbate

**Affiliations:** ^1^Department of Political Science and International Relations (DEMS), University of Palermo, Palermo, Italy; ^2^Atkinson Graduate School of Management, Willamette University, Salem, OR, United States; ^3^Department of Psychology, Educational Science and Human Movement, University of Palermo, Palermo, Italy

**Keywords:** choice-overload, decision-making, choice set complexity, decision task difficulty, decision goal, decision-making tendency

## Abstract

Researchers investigating the psychological effects of choice have provided extensive empirical evidence that having choice comes with many advantages, including better performance, more motivation, and greater life satisfaction and disadvantages, such as avoidance of decisions and regret. When the decision task difficulty exceeds the natural cognitive resources of human mind, the possibility to choose becomes more a source of unhappiness and dissatisfaction than an opportunity for a greater well-being, a phenomenon referred to as choice overload. More recently, internal and external moderators that impact when choice overload occurs have been identified. This paper reviews seminal research on the advantages and disadvantages of choice and provides a systematic qualitative review of the research examining moderators of choice overload, laying out multiple critical paths forward for needed research in this area. We organize this literature review using two categories of moderators: the choice environment or context of the decision as well as the decision-maker characteristics.

## Introduction

The current marketing orientation adopted by many organizations is to offer a wide range of options that differ in only minor ways. For example, in a common western grocery store contains 285 types of cookies, 120 different pasta sauces, 175 salad-dressing, and 275 types of cereal (Botti and Iyengar, 2006). However, research in psychology and consumer behavior has demonstrated that when the number of alternatives to choose from becomes excessive (or superior to the decision-makers’ cognitive resources), choice is mostly a disadvantage to both the seller and the buyer. This phenomenon has been called *choice overload* and it refers to a variety of negative consequences stemming from having too many choices, including increased choice deferral, switching likelihood, or decision regret, as well as decreased choice satisfaction and confidence (e.g., Chernev et al., 2015). Choice overload has been replicated in numerous fields and laboratory settings, with different items (e.g., jellybeans, pens, coffee, chocolates, etc.), actions (reading, completing projects, and writing essays), and populations (e.g., Chernev, 2003; Iyengar et al., 2004; Schwartz, 2004; Shah and Wolford, 2007; Mogilner et al., 2008; Fasolo et al., 2009; Misuraca and Teuscher, 2013; Misuraca and Faraci, 2021; Misuraca et al., 2022; see also Misuraca, 2013). Over time, we have gained insight into numerous moderators of the choice overload phenomena, including aspects of the context or choice environment as well as the individual characteristics of the decision-maker (for a detailed review see Misuraca et al., 2020).

The goal of this review is to summarize important research findings that drive our current understanding of the advantages and disadvantages of choice, focusing on the growing body of research investigating moderators of choice overload. Following a discussion of the advantages and disadvantages of choice, we review the existing empirical literature examining moderators of choice overload. We organize this literature review using two categories of moderators: the choice environment or context of the decision as well as the decision-maker characteristics. Finally, based on this systematic review of research, we propose a variety of future research directions for choice overload investigators, ranging from exploring underlying mechanisms of choice overload moderators to broadening the area of investigation to include a robust variety of decision-making scenarios.

## Theoretical background

### The advantages of choice

Decades of research in psychology have demonstrated the many advantages of choice. Indeed, increased choice options are associated with increase intrinsic motivation (Deci, 1975; Deci et al., 1981; Deci and Ryan, 1985), improved task performance (Rotter, 1966), enhanced life satisfaction (Langer and Rodin, 1976), and improved well-being (Taylor and Brown, 1988). Increased choice options also have the potential to satisfy heterogeneous preferences and produce greater utility (Lancaster, 1990). Likewise, economic research has demonstrated that larger assortments provide a higher chance to find an option that perfectly matches the individual preferences (Baumol and Ide, 1956). In other words, with larger assortments it is easier to find what a decision-maker wants.

The impact of increased choice options extends into learning, internal motivation, and performance. Zuckerman et al. (1978) asked college students to solve puzzles. Half of the participants could choose the puzzle they would solve from six options. For the other half of participants, instead, the puzzle was imposed by the researchers. It was found that the group free to choose the puzzle was more motivated, more engaged and exhibited better performance than the group that could not choose the puzzle to solve. In similar research, Schraw et al. (1998) asked college students to read a book. Participants were assigned to either a choice condition or a non-choice condition. In the first one, they were free to choose the book to read, whereas in the second condition the books to read were externally imposed, according to a yoked procedure. Results demonstrated the group that was free to make decisions was more motivated to read, more engaged, and more satisfied compared to the group that was not allowed to choose the book to read (Schraw et al., 1998).

These effects remain consistent with children and when choice options are constrained to incidental aspects of the learning context. In the study by Cordova and Lepper (1996), elementary school children played a computer game designed to teach arithmetic and problem-solving skills. One group could make decisions about incidental aspects of the learning context, including which spaceship was used and its name, whereas another group could not make any choice (all the choices about the game’s features were externally imposed by the experimenters). The results demonstrated that the first group was more motivated to play the game, more engaged in the task, learned more of the arithmetical concepts involved in the game, and preferred to solve more difficult tasks compared to the second group.

Extending benefits of choice into health consequences, Langer and Rodin (1976) examined the impact that choice made in nursing home patients. In this context, it was observed that giving patients the possibility to make decisions about apparently irrelevant aspects of their life (e.g., at what time to watch a movie; how to dispose the furniture in their bedrooms, etc.), increased psychological and physiological well-being. The lack of choice resulted, instead, in a state of learned helplessness, as well as deterioration of physiological and psychological functions.

The above studies lead to the conclusion that choice has important advantages over no choice and, to some extent, limited choice options. It seems that providing more choice options is an improvement – it will be more motivating, more satisfying, and yield greater well-being. In line with this conclusion, the current orientation in marketing is to offer a huge variety of products that differ only in small details (e.g., Botti and Iyengar, 2006). However, research in psychology and consumer behavior demonstrated that when the number of alternatives to choose from exceeds the decision-makers’ cognitive resources, choice can become a disadvantage.

### The disadvantages of choice

A famous field study conducted by Iyengar and Lepper (2000) in a Californian supermarket demonstrated that too much choice decreases customers’ motivation to buy as well as their post-choice satisfaction. Tasting booths were set up in two different areas of the supermarket, one of which displayed 6 different jars of jam while the other displayed 24 options, with customers free to taste any of the different flavors of jam. As expected, the larger assortment attracted more passers-by compared to the smaller assortment; Indeed, 60% of passers-by stopped at the table displaying 24 different options, whereas only 40% of the passers-by stopped at the table displaying the small variety of 6 jams. This finding was expected given that more choice options are appealing. However, out of the 60% of passers-by who stopped at the table with more choices, only 3% of them decided to buy jam. Conversely, 30% of the consumers who stopped at the table with only 6 jars of jam decided to purchase at least one jar. Additionally, these customers expressed a higher level of satisfaction with their choices, compared to those who purchased a jar of jam from the larger assortment. In other words, it seems that too much choice is at the beginning more appealing (attracts more customers), but it decreases the motivation to choose and the post-choice satisfaction.

This classic and seminal example of choice overload was quickly followed by many replications that expanded the findings from simple purchasing decisions into other realms of life. For example, Iyengar and Lepper (2000), asked college students to write an essay. Participants were randomly assigned to one of the following two experimental conditions: limited-choice condition, in which they could choose from a list of six topics for the essay, and extensive-choice condition, in which they could choose from a list of 30 different topics for the essay. Results showed that a higher percentage of college students (74%) turned in the essay in the first condition compared to the second condition (60%). Moreover, the essays written by the students in the limited-choice conditions were evaluated as being higher quality compared to the essays written by the students in the extensive choice condition. In a separate study, college students were asked to choose one chocolate from two randomly assigned choice conditions with either 6 or 30 different chocolates. Those participants in the limited choice condition reporting being more satisfied with their choice and more willing to purchase chocolates at the end of the experiment, compared to participants who chose from the larger assortment (Iyengar and Lepper, 2000).

In the field of financial decision-making, Iyengar et al. (2004) analyzed 800,000 employees’ decisions about their participation in 401(k) plans that offered from a minimum of 2 to a maximum of 59 different fund options. The researchers observed that as the fund options increased, the participation rate decreased. Specifically, plans offering less than 10 options had the highest participation rate, whereas plans offering 59 options had the lowest participation rate.

The negative consequences of having too much choice driven by cognitive limitations. Simon (1957) noted that decision-makers have a bounded rationality. In other words, the human mind cannot process an unlimited amount of information. Individuals’ working memory has a span of about 7 (plus or minus two) items (Miller, 1956), which means that of all the options to choose from, individuals can mentally process only about 7 alternatives at a time. Because of these cognitive limitations, when the number of choices becomes too high, the comparison of all the available items becomes cognitively unmanageable and, consequently, decision-makers feel overwhelmed, confused, less motivated to choose and less satisfied (e.g., Iyengar and Lepper, 2000). However, a more recent meta-analytic work [Chernev et al., 2015: see also Misuraca et al. (2020)] has shown that choice overload occurs only under certain conditions. Many moderators that mitigate the phenomenon have been identified by researchers in psychology and consumer behavior (e.g., Mogilner et al., 2008; Misuraca et al., 2016a). In the next sections, we describe our review methodology and provide a detailed discussion of the main external and internal moderators of choice overload.

## Methods

### Literature search and inclusion criteria

Our investigation consisted of a literature review of peer-reviewed empirical research examining moderators of choice overload. We took several steps to locate and identify eligible studies. First, we sought to establish a list of moderators examined in the choice overload literature. For this, we referenced reviews conducted by Chernev et al. (2015), McShane and Böckenholt (2017), as well as Misuraca et al. (2020) and reviewed the references sections of the identified articles to locate additional studies. Using the list of moderators generated from this examination, we conducted a literature search using PsycInfo (Psychological Abstracts), EBSCO and Google Scholar. This search included such specific terms such as choice set complexity, visual preference heuristic, and choice preference uncertainty, as well as broad searches for ‘choice overload’ and ‘moderator’.

We used several inclusion criteria to select relevant articles. First, the article had to note that it was examining the choice overload phenomena. Studies examining other theories and/or related variables were excluded. Second, to ensure that we were including high-quality research methods that have been evaluated by scholars, only peer-reviewed journal articles were included. Third, the article had to include primary empirical data (qualitative or quantitative). Thus, studies that were conceptual in nature were excluded. This process yielded 49 articles for the subsequent review.

## Moderators of choice overload

### Choice environment and context

Regarding external moderators of choice overload, several aspects about the choice environment become increasingly relevant. Specifically, these include the perceptual attributes of the information, complexity of the set of options, decision task difficulty, as well as the presence of brand names.

#### Perceptual characteristics

As Miller (1956) noted, humans have “channel capacity” for information processing and these differ for divergent stimuli: for taste, we have a capacity to accommodate four; for tones, the capacity increased to six; and for visual stimuli, we have the capacity for 10–15 items. Accordingly, perceptual attributes of choice options are an important moderator of choice overload, with visual presentation being one of the most important perceptual attributes (Townsend and Kahn, 2014). The *visual preference heuristic* refers to the tendency to prefer a visual rather than verbal representation of choice options, regardless of assortment size (Townsend and Kahn, 2014). However, despite this preference, visual presentations of large assortments lead to suboptimal decisions compared to verbal presentations, as visual presentations activate a less systematic decision-making approach (Townsend and Kahn, 2014). Visual presentation of large choice sets is also associated with increased perceptions of complexity and likelihood of decisions deferral. Visual representations are particularly effective with small assortments, as they increase consumers’ perception of variety, improve the likelihood of making a choice, and reduce the time spent examining options (Townsend and Kahn, 2014).

#### Choice set complexity

Choice set complexity refers to a wide range of aspects of a decision task that affect the value of the available choice options without influencing the structural characteristics of the decision problem (Payne et al., 1993). Thus, choice set complexity does not influence aspects such as the number of options, number of attributes of each option, or format in which the information is presented. Rather, choice set complexity concerns factors such as the attractiveness of options, the presence of a dominant option, and the complementarity or alignability of the options.

Choice set complexity increases when the options include higher-quality, more attractive options (Chernev and Hamilton, 2009). Indeed, when the variability in the relative attractiveness of the choice alternatives increases, the certainty about the choice and the satisfaction with the task increase (Malhotra, 1982). Accordingly, when the number of attractive options increases, more choice options led to a decline in consumer satisfaction and likelihood of a decision being made, but satisfaction increases and decision deferral decreased when the number of unattractive options increases (Dhar, 1997). This occurs when increased choice options make the weakness and strengths of attractive and unattractive options more salient (Chan, 2015).

Similarly, the presence of a dominant option simplifies large choice sets and increased the preference for the chosen option; however, the opposite effect happens in small choice sets (Chernev, 2003). Choice sets containing an ideal option have been associated with increased brain activity in the areas involved in reward and value processing as well as in the integration of costs and benefits (striatum and the anterior cingulate cortex; Reutskaja et al., 2018) which could explain why larger choice sets are not always associated with choice overload. As Misuraca et al. (2020, p. 639) noted, “*the benefits of having an ideal item in the set might compensate for the costs of overwhelming set size in the bounded rational mind of humans*.*”*

Finally, choice set complexity is impacted by the alignability and complementarity of the attributes that differentiate the options (Chernev et al., 2015). When unique attributes of options exist within a choice set, complexity and choice overload increase as the unique attributes make comparison more difficult and trade-offs more salient. Indeed, feature alignability and complementarity (meaning that the options have additive utility and need to be co-present to fully satisfy the decision-maker’s need)^1^ have been associated with decision deferral (Chernev, 2005; Gourville and Soman, 2005) and changes in satisfaction (Griffin and Broniarczyk, 2010).

#### Decision task difficulty

Decision task difficulty refers to the structural characteristics of a decision problem; unlike choice set complexity, decision task difficulty does not influence the value of the choice options (Payne et al., 1993). Decision task difficulty is influenced by the number of attributes used to describe available options, decision accountability, time constraints, and presentation format.

The number of attributes used to describe the available options within an assortment influences decision task difficulty and choice overload (Hoch et al., 1999; Chernev, 2003; Greifeneder et al., 2010), such that choice overload increases with the number of dimensions upon which the options differ. With each additional dimension, decision-makers have another piece of information that must be attended to and evaluated. Along with increasing the cognitive complexity of the choice, additional dimensions likely increase the odds that each option is inferior to other options on one dimension or another (e.g., Chernev et al., 2015).

When individuals have decision accountability or are required to justify their choice of an assortment to others, they tend to prefer larger assortments; However, when individuals must justify their particular choice from an assortment to others, they tend to prefer smaller choice sets (Ratner and Kahn, 2002; Chernev, 2006; Scheibehenne et al., 2009). Indeed, decision accountability is associated with decision deferral when choice sets are larger compared to smaller (Gourville and Soman, 2005). Thus, decision accountability influences decision task difficulty differently depending on whether an individual is selecting an assortment or choosing an option from an assortment.

Time pressure or constraint is an important contextual factor for decision task difficulty, choice overload, and decision regret (Payne et al., 1993). Time pressure affects the strategies that are used to make decisions as well as the quality of the decisions made. When confronted with time pressure, decision-makers tend to speed up information processing, which could be accomplished by limiting the amount of information that they process and use (Payne et al., 1993; Pieters and Warlop, 1999; Reutskaja et al., 2011). Decision deferral becomes a more likely outcome, as is choosing at random and regretting the decision later (Inbar et al., 2011).

The physical arrangement and presentation of options and information affect information perception, processing, and decision-making. This moderates the effect of choice overload because these aspects facilitate or inhibit decision-makers’ ability to process a greater information load (e.g., Chernev et al., 2015; Anderson and Misuraca, 2017). The location of options and structure of presented information allow the retrieval of information about the options, thus allowing choosers to distinguish and evaluate various options (e.g., Chandon et al., 2009). Specifically, organizing information into “chunks” facilitates information processing (Miller, 1956) as well as the perception of greater variety in large choice sets (Kahn and Wansink, 2004). Interestingly, these “chunks” do not have to be informative; Mogilner et al. (2008) found that choice overload was mitigated to the same extent when large choice sets were grouped into generic categories (i.e., A, B, etc.) as when the categories were meaningful descriptions of characteristics.

Beyond organization, the presentation order can facilitate or inhibit decision-makers cognitive processing ability. Levav et al. (2010) found that choice overload decreased and choice satisfaction increased when smaller choice sets were followed by larger choice sets, compared to the opposite order of presentation. When sets are highly varied, Huffman and Kahn (1998) found that decision-makers were more satisfied and willing to make a choice when information was presented about attributes (i.e., price and characteristics) rather than available alternatives (i.e., images of options). Finally, presenting information simultaneously, rather than sequentially, increases decision satisfaction (Mogilner et al., 2013), likely due to decision-makers choosing among an available set rather than comparing each option to an imaged ideal option.

#### Brand names

The presence of brand names is an important moderator of choice overload. As recently demonstrated by researchers in psychology and consumer behavior, choice overload occurs only when options are not associated with brands, choice overload occurs when the same choice options are presented without any brand names (Misuraca et al., 2019, 2021a). When choosing between 6 or 24 different mobile phones, choice overload did not occur in the condition in which phones were associated with a well-known brand (i.e., Apple, Samsung, Nokia, etc.), although it did occur when the same cell phones were displayed without information about their brand. These findings have been replicated with a population of adolescents (Misuraca et al., 2021a).

### Decision-maker characteristics

Beyond the choice environment and context, individual differences in decision-maker characteristics are significant moderators of choice overload. Several critical characteristics include the decision goal as well as an individual’s preference uncertainty, affective state, decision style, and demographic variables such as age, gender, and cultural background (e.g., Misuraca et al., 2021a).

#### Decision goal

A decision goal refers to the extent to which a decision-maker aims to minimize the cognitive resources spent making a decision (Chernev, 2003). Decision goals have been associated with choice overload, with choice overload increasing along with choice set options, likely due to decision-makers unwillingness to make tradeoffs between various options. As a moderator of choice overload, there are several factors which impact the effect of decision goals, including decision intent (choosing or browsing) and decision focus (choosing an assortment or an option) (Misuraca et al., 2020).

Decision intent varies between choosing, with the goal of making a decision among the available options, and browsing, with the goal of learning more about the options. Cognitive overload is more likely to occur than when decision makers’ goal is choosing compared to browsing. For choosing goals, decision-makers need to make trade-offs among the pros and cons of the options, something that demands more cognitive resources. Accordingly, decision-makers whose goal is browsing, rather than choosing, are less likely to experience cognitive overload when facing large assortments (Chernev and Hamilton, 2009). Furthermore, when decision-makers have a goal of choosing, brain research reveals inverted-U-shaped function, with neither too much nor too little choice providing optimal cognitive net benefits (Reutskaja et al., 2018).

Decision focus can target selecting an assortment or selecting an option from an assortment. When selecting an assortment, cognitive overload is less likely to occur, likely due to the lack of individual option evaluation and trade-offs (Chernev et al., 2015). Thus, when choosing an assortment, decision-makers tend to prefer larger assortments that provide more variety. Conversely, decision-makers focused on choosing an option from an assortment report increased decision difficulty and tend to prefer smaller assortments (Chernev, 2006). Decision overload is further moderated by the order of decision focus. Scheibehenne et al. (2010) found that when decision-makers first decide on an assortment, they are more likely to choose an option from that assortment, rather than an option from an assortment they did not first select.

#### Preference uncertainty

The degree to which decision-makers have preferences varies regarding comprehension and prioritization of the costs and benefits of the choice options. This is referred to as preference uncertainty (Chernev, 2003). Preference uncertainty is influenced by decision-maker expertise and an articulated ideal option, which indicates well-defined preferences. When decision-makers have limited expertise, larger choice sets are associated with weaker preferences as well as increased choice deferral and choice overload compared to smaller choice sets. Conversely, high expertise decision-makers experience weaker preferences and increased choice deferral in the context of smaller choice sets compared to larger (Mogilner et al., 2008; Morrin et al., 2012). Likewise, an articulated ideal option, which implies that the decision-maker has already engaged in trade-offs, is associated with reduced decision complexity. The effect is more pronounced in larger choice sets compared to smaller choice sets (Chernev, 2003).

#### Positive affect

Positive affect tends to moderate the impact of choice overload on decision satisfaction. Indeed, Spassova and Isen (2013) found that decision-makers reporting positive affect did not report experiencing dissatisfaction when choosing from larger choice sets while those with neutral affect reported being more satisfied when choosing from smaller choice sets. This affect may be associated with the affect heuristic, or a cognitive shortcut that enables efficient decisions based on the immediate emotional response to a stimulus (Slovic et al., 2007).

#### Decision-making tendencies

Satisfaction with extensive choice options may depend on whether one is a maximizer or a satisficer. Maximizing refers to the tendency to search for the best option. Maximizers approach decision tasks with the goal to find the absolute best (Carmeci et al., 2009; Misuraca et al., 2015, 2016b, 2021b; Misuraca and Fasolo, 2018). To do that, they tend to process all the information available and try to compare all the possible options. Conversely, satisficers are decision-makers whose goal is to select an option that is good enough, rather than the best choice. To find such an option, satisficers evaluate a smaller range of options, and choose as soon as they find one alternative that surpasses their threshold of acceptability (Schwartz, 2004). Given the different approach of maximizers and satisficers when choosing, it is easy to see why choice overload represents more of a problem for maximizers than for satisficers. If the number of choices exceeds the individuals’ cognitive resources, maximizers more than satisficers would feel overwhelmed, frustrated, and dissatisfied, because an evaluation of all the available options to select the best one is cognitively impossible.

Maximizers attracted considerable attention from researchers because of the paradoxical finding that even though they make objectively better decisions than satisficers, they report greater regret and dissatisfaction. Specifically, Iyengar et al. (2006), analyzed the job search outcomes of college students during their final college year and found that maximizer students selected jobs with 20% higher salaries compared to satisficers, but they felt less satisfied and happy, as well as more stressed, frustrated, anxious, and regretful than students who were satisficers. The reasons for these negative feelings of maximizers lies in their tendency to believe that a better option is among those that they could not evaluate, given their time and cognitive limitations.

#### Choosing for others versus oneself

When decision-makers must make a choice for someone else, choice overload does not occur (Polman, 2012). When making choices for others (about wines, ice-cream flavors, school courses, etc.), decision makers reported greater satisfaction when choosing from larger assortments rather than smaller assortments. However, when choosing for themselves, they reported higher satisfaction after choosing from smaller rather than larger assortments.

#### Demographics

Demographic variables such as gender, age, and cultural background moderate reactions concerning choice overload. Regarding gender, men and women may often employ different information-processing strategies, with women being more likely to attend to and use details than men (e.g., Meyers-Levy and Maheswaran, 1991). Gender differences also arise in desire for variety and satisfaction depending on choice type. While women were more satisfied with their choice of gift boxes regardless of assortment size, women become more selective than men when speed-dating with larger groups of speed daters compared to smaller groups (Fisman et al., 2006).

Age moderates the choice overload experience such that, when choosing from an extensive array of options, adolescents and adults suffer similar negative consequences (i.e., greater difficulty and dissatisfaction), while children and seniors suffer fewer negative consequences (i.e., less difficulty and dissatisfaction than adolescents and adults) (Misuraca et al., 2016a). This could be associated with decision-making tendencies. Indeed, adults and adolescents tend to adopt maximizing approaches (Furby and Beyth-Marom, 1992). This maximizing tendency aligns with their greater perceived difficulty and post-choice dissatisfaction when facing a high number of options (Iyengar et al., 2006). Seniors tend to adopt a satisficing approach when making decisions (Tanius et al., 2009), as well as become overconfident in their judgments (Stankov and Crawford, 1996) and focused on positive information (Mather and Carstensen, 2005). Taken together, these could explain why the negative consequences of too many choice options were milder among seniors. Finally, children tend to approach decisions in an intuitive manner and quickly develop strong preferences (Schlottmann and Wilkening, 2011). This mitigates the negative consequences of choice overload for this age group.

Finally, decision-makers from different cultures have different preferences for variety (e.g., Iyengar, 2010). Eastern Europeans report greater satisfaction with larger choice sets than Western Europeans (Reutskaja et al., 2022). Likewise, cultural differences in perception may impact how choice options affect decision-makers from Western and non-Western cultures (e.g., Miyamoto et al., 2006).

## Future research directions

As researchers continue to investigate the choice overload phenomenon, future investigations can provide a deeper understanding of the underlying mechanisms that influence when and how individuals experience the negative impacts of choice overload as well as illuminate how this phenomenon can affect people in diverse contexts (such as hiring decisions, sports, social media platforms, streaming services, etc.).

For instance, the visual preference heuristic indicates, and subsequent research supports, the human tendency to prefer visual rather than verbal representations of choice options (Townsend and Kahn, 2014). However, in Huffman and Kahn’s (1998) research, decision-makers preferred written information, such as characteristics of the sofa, rather than visual representations of alternatives. Future researchers can investigate the circumstances that underlie when individuals prefer detailed written or verbal information as opposed to visual images.

Furthermore, future researchers can examine the extent to which the mechanisms underlying the impact of chunking align with those underlying the effect of brand names. Research has supported that chunking information reduces choice overload, regardless of the sophistication of the categories (Kahn and Wansink, 2004; Mogilner et al., 2008). The presence of a brand name has a seemingly similar effect (Misuraca et al., 2019, 2021a). The extent to which the cognitive processes underlying these two areas of research the similar, as well as the ways in which they might differ, can provide valuable insights for researchers and practitioners.

More research is needed that considers the role of the specific culture and cultural values of the decision-maker on choice overload. Indeed, the traditional studies on the choice overload phenomenon mentioned above predominantly focused on western cultures, which are known for being individualistic cultures. Future research should explore whether choice overload replicates in collectivistic cultures, which value the importance of making personal decisions differently than individualist cultures. Additional cultural values, such as long-term or short-term time orientation, may also impact decision-makers and the extent to which they experience choice overload (Hofstede and Minkov, 2010).

While future research that expands our understanding of the currently known and identified moderators of choice overload can critically inform our understanding of when and how this phenomenon occurs, there are many new and exciting directions into which researchers can expand.

For example, traditional research on choice overload focused on choice scenarios where decision-makers had to choose only one option out of either a small or a large assortment of options. This is clearly an important scenario, yet it represents only one of many scenarios that choice overload may impact. Future research could investigate when and how this phenomenon occurs in a wide variety of scenarios that are common in the real-world but currently neglected in classical studies on choice overload. These could include situations in which the individual can choose more than one option (e.g., more than one type of ice cream or cereal) (see Fasolo et al., 2024).

Historically, a significant amount of research on choice overload has focused on purchasing decisions. Some evidence also indicates that the phenomenon occurs in a variety of situations (e.g., online dating, career choices, retirement planning, travel and tourism, and education), potentially hindering decision-making processes and outcomes. Future research should further investigate how choice overload impacts individuals in a variety of untested situations. For instance, how might choice overload impact the hiring manager with a robust pool of qualified applicants? How would the occurrence of choice overload in a hiring situation impact the quality of the decision, making an optimal hire? Likewise, does choice overload play a role in procrastination? When confronted with an overwhelming number of task options, does choice overload play a role in decision deferral? It could be that similar cognitive processes underlie deferring a choice on a purchase and deferring a choice on a to-do list. Research is needed to understand how choice overload (and its moderators) may differ across these scenarios.

Finally, as society continues to adapt and develop, future research will be needed to evaluate the impact these technological and sociological changes have on individual decision-makers. The technology that we interact with has become substantially more sophisticated and omnipresent, particularly in the form of artificial intelligence (AI). As AI is adopted into our work, shopping, and online experiences, future researchers should investigate if AI and interactive decision-aids (e.g., Anderson and Misuraca, 2017) can be effectively leveraged to reduce the negative consequences of having too many alternatives without impairing the sense of freedom of decision-makers.

As with technological advancements, future research could examine how new sociological roles contribute to or minimize choice overload. For example, a social media influencer could reduce the complexity of the decision when there is a large number of choice options. If social media influencers have an impact, is that impact consistent across age groups and culturally diverse individuals? Deepening our understanding of how historical and sociological events have impacted decision-makers, along with how cultural differences in our perceptions of the world as noted above, could provide a rich and needed area of future research.

## Discussion and conclusion

Research in psychology demonstrated the advantages of being able to make choices from a variety of alternatives, particularly when compared to no choice at all. Having the possibility to choose, indeed, enhances individuals’ feeling of self-determination, motivation, performance, well-being, and satisfaction with life (e.g., Zuckerman et al., 1978; Cordova and Lepper, 1996). As the world continues to globalize through sophisticated supply chains and seemingly infinite online shopping options, our societies have become characterized by a proliferation of choice options. Today, not only stores, but universities, hospitals, financial advisors, sport centers, and many other businesses offer a huge number of options from which to choose. The variety offered is often so large that decision-makers can become overwhelmed when trying to compare and evaluate all the potential options and experience choice overload (Iyengar and Lepper, 2000). Rather than lose the benefits associated with choice options, researchers and practitioners should understand and leverage the existence of the many moderators that affect the occurrence of choice overload. The findings presented in this review indicate that choice overload is influenced by several factors, including perceptual attributes, choice set complexity, decision task difficulty, and brand association. Understanding these moderators can aid in designing choice environments that optimize decision-making processes and alleviate choice overload. For instance, organizing options effectively and leveraging brand association can enhance decision satisfaction and reduce choice overload. Additionally, considering individual differences such as decision goals, preference uncertainty, affective state, decision-making tendencies, and demographics can tailor decision-making environments to better suit the needs and preferences of individuals, ultimately improving decision outcomes. Future research is needed to fully understand the role of many variables that might be responsible for the negative consequences of choice overload and to better understand under which conditions the phenomenon occurs.

## Author contributions

RM: Writing – review & editing, Conceptualization, Data curation, Investigation, Methodology, Writing – original draft. AN: Writing – review & editing. SM: Writing – review & editing. GD: Methodology, Writing – review & editing. CS: Writing – review & editing, Supervision.

## Footnotes

^1^For example, gloves and socks have complementary features, in that they provide warmth to different parts of the body.
